# Effect of 4 years of seasonal malaria chemoprevention on the acquisition of antibodies to *Plasmodium falciparum* antigens in Ouelessebougou, Mali

**DOI:** 10.1186/s12936-020-03542-9

**Published:** 2021-01-07

**Authors:** Almahamoudou Mahamar, Djibrilla Issiaka, Ahamadou Youssouf, Sidi M. Niambele, Harouna M. Soumare, Oumar Attaher, Amadou Barry, David L. Narum, Patrick E. Duffy, Brian Greenwood, Michal Fried, Alassane Dicko

**Affiliations:** 1 Mali Research & Training Center, Faculty of Medicine, Pharmacy and Dentistry, University of Science, Techniques and Technologies (USTT), Bamako, Mali; 2 Laboratory of Malaria Immunology and Vaccinology (LMIV), National Institute of Allergy and Infectious Diseases (NIAID), National Institutes of Health (NIH), Rockville, MD USA; 3 London School of Hygiene and Tropical Medicine, Keppel St, London, WC1E 7HT UK

**Keywords:** Antibody to MSP-1_42_, AMA1, CSP, Seasonal malaria chemoprevention, Seropositivity

## Abstract

**Background:**

More than 200 million people live in areas of highly seasonal malaria transmission where Seasonal Malaria Chemoprevention (SMC) with sulfadoxine-pyrimethamine (SP) and amodiaquine (AQ) was recommended in 2012 by WHO. This strategy is now implemented widely and protected more than 19 million children in 2018. It was previously reported that exposure to SMC reduced antibody levels to AMA1, MSP-1_42_ and CSP, but the duration of exposure to SMC up to three 3 years, had no effect on antibody levels to MSP-1_42_ and CSP.

**Methods:**

In 2017, a cross-sectional survey was carried out 1 month after the last dose of SMC had been given to children aged 4–5 years randomly selected from areas where SMC had been given for 2 or 4 years during the malaria transmission season. A total of 461 children were enrolled, 242 children in areas where SMC had been implemented for 4 years and 219 children in areas where SMC had been implemented for 2 years. Antibody extracted from dry blood spots was used to measure IgG levels to the malaria antigens CSP, MSP-1_42_ and AMA1 by ELISA.

**Results:**

The prevalence of antibodies to MSP-1_42_ was similar in children who had received SMC for 4 years compared to those who had received SMC for only 2 years (85.1 vs 86.0%, ajusted odd ratio (aOR) = 1.06, 95% confidence intervals (CI 0.62–1.80), p = 0.80). The prevalence of antibodies to AMA-1 and to CSP was not lower in children who received SMC for 4 years compared to those who had received SMC for only 2 years (95.3 vs 88.8%, aOR = 3.16, 95% CI 1.44–6.95, p = 0.004 for AMA-1; and 91.2 vs 81.9%, aOR = 3.14, 95% CI 1.70–5.76, p < 0.001 for CSP). Median antibody levels for anti-MSP-1_42_ IgG were not significatively inferior in children who had received SMC for four rather than 2 years (0.88 (IQR: 0.64–1.15) and 0.95 ((0.68–1.15), respectively), anti-CSP (1.30 (1.00–1.56) and 1.17 (0.87–1.47)), and anti-AMA-1 (1.45 (1.24–1.68) and 1.41 (1.17–1.64)).

**Conclusion:**

In an area of high seasonal malaria transmission, children who had received SMC for 4 years did not had lower seropositivity or antibody levels to AMA1, MSP-1_42_ and CSP compared to children who had received SMC for only 2 years suggesting that children who have received SMC for 4 years may not be more at risk of malaria after the cessation of SMC than children who have received SMC for a shorter period.

## Background

The health information system in Mali reported that in 2018 malaria was responsible for 32% of all outpatient consultations in health facilities with 2.34 million clinical cases of malaria [1]. Since 2012, Seasonal Malaria Chemoprevention (SMC) has been recommended by the World Health Organization (WHO) for children aged 3–59 months living in areas of highly seasonal malaria transmission in this sub-region [2]. SMC consists of full treatment courses of sulfadoxine–pyrimethamine plus amodiaquine (SP + AQ) given to children 3–59 months of age, at monthly intervals during the malaria transmission season to maintain therapeutic anti-malarial drug concentrations in the blood throughout the period of greatest malaria risk [2]. In 2018, 19 million children in 12 countries in Africa’s Sahel sub-region were protected through SMC programmes [3].

Despite the substantial benefits provided by SMC, one area of concern is that SMC will impair the natural acquisition of protective immune responses, thereby increasing the risk of disease in later years. Serological markers can be used to monitoring malaria immunity in areas where control intervention efforts have been undertaken. They also provide useful baseline information about the intensity of malaria transmission in different epidemiological situations.

Early studies of SMC showed small increases in clinical malaria following the cessation of a single year of the intervention in Mali and Burkina Faso [4, 5]. In Mozambique, antibody levels to *Plasmodium falciparum* erythrocytic-stage antigens in the first 2 years of life were not different after chemoprophylaxis with SP administered at 3, 4 and 9 months of age [6]. In Senegal, seropositivity to the blood-stage antigens AMA-1 and GLURP were higher in children in areas where SMC was not implemented compared to those in the area where it was implemented although there was no significant difference between children who did or did not receive SMC in the area where the strategy was implemented [7]. In a high-transmission setting in Uganda, children randomly assigned to receive monthly chemoprevention with dihydroartemisinin-piperaquine had a greater percentage of infected red blood cells specific CD4 + T cells coproducing IL-2 and TNF, which were associated with protection from subsequent malaria and parasitaemia, and fewer CD4 + T cells coproducing IL-10/ IFN-γ, which were associated with an increased risk of malaria than control children [8]. A lower prevalence of seropositivity for MSP-1_19_ was reported in children, who received biannual azithromycin preventive treatment compared to whose who received annual treatment in Niger [9].

In a recent study in Ouelessebougou, an area of high seasonal malaria transmission in Mali, the measurement of seroprevalence was less sensitive than measuring IgG levels in detecting differences between children who did or did not receive SMC. Exposure to SMC reduced antibody levels to AMA1, MSP-1_42_ and CSP, but the duration of exposure to SMC up to three 3 years, had no effect on antibody levels to MSP-1_42_ and CSP [10]. The study compared findings in children who never received SMC and those who received it for one, two or three years. In the study reported in this paper, the impact of 4 years of SMC compared to 2 years of SMC on the acquisition of antibodies to malaria antigens was assessed. Seropositivity and antibody levels to pre-erythrocytic stage (CSP) and blood stage (MSP-1_42_, AMA1) antigens were measured in the two groups and compared.

## Methods

### Study site and procedure

The study was conducted in the health district of Ouelessebougou, located 80 km south of Bamako, Mali. The target population was children aged 3 to 59 months of age. SMC was implemented progressively in the district of Ouelessebougou as described previously [10]. Briefly, eight of the 13 sub-districts of Ouelessebougou were randomly selected to receive SMC progressively each year, four in 2014 (year 1) two in 2015 (year 2) and two in 2016 (year 3). In 2016, SMC was extended to the entire district by the National Malaria Control Programme (NMCP). Children in the selected areas received three rounds of SMC in the first year, and four rounds of SMC in the following years. Doses of SMC were given per age according to the WHO recommendation. Children aged 3–11 months received 75 mg of AQ given once daily for 3 days plus a single dose of 250/12.5 mg of SP, while children aged 12–59 months received 150 mg AQ base given once daily for 3 days and a single dose of 500/25 mg of SP. The single dose of SP was given on the first day at the same time as the first dose of AQ. SMC was administrated during the peak malaria transmission at monthly interval for 3 months in 2014 and 4 months the following years. At each cycle the proportion of children who received SMC was estimated to 77 to 90% [11].

To assess the effect of SMC on the acquisition of antibodies to *P. falciparum* antigens, a cross-sectional survey was conducted at the end of malaria transmission season in January 2018 about 6–8 weeks after the last SMC cycle in children aged 4–5 years. These children were randomly selected from the population in areas where SMC was given for 2 or 4 years using a two-stage sampling. First, ten villages were randomly selected from these areas where SMC was implemented for 2 or 4 years and second, children in the target age ranges were randomly selected using the census lists of children in these villages. After obtaining informed consent, finger prick blood samples were obtained for collection of a blood smear and filter paper blood spots (Whatman^®^ protein saver cards, Z761575 ALDRICH). Dried filter papers were stored at − 20° until use.

### Laboratory methods

#### Recombinant proteins

All samples were assayed for Immunoglobulin G to recombinant proteins. The plasmodial antigens used in this study included recombinant AMA-1 (3D7 strain) [12] and MSP-1_42_ (FVO strain) expressed in *Escherichia coli* [13] and recombinant *P*. *falciparum* 3D7 CSP clone expressed in *Pichia pastoris* [14].

#### Antibody determination by ELISA

IgG was eluted from dried filter papers as previously described [10, 15]. Briefly, two filter paper discs of 2.5 mm in diameter were taken from the center of a single dried bloodspot and added into a deep well plate, incubated in 1120 μL of a 0.5% saponin solution at room temperature (RT). Plates were sealed and placed onto a plate shaker overnight. High-binding 96-well Immulon HBX4 microplates were coated with 200 ng per well of antigen diluted in 0.05 M carbonate-bicarbonate buffer and incubated overnight at 4 °C. The final serum dilution of the eluate based on estimates of the volume of whole blood in a 2.5 mm filter paper disc was 1:400 [15]. Blank, positive control (hyperimmune serum pool) and negative control (American malaria-naive controls) wells diluted 1:400 were included in each plate in duplicate at the same concentration as the test samples. Plates were blocked with 5% skim milk in PBS for 1 h 30 min at room temperature. Plates were washed with 0.05% Tween 20 in PBS (PBS-Tween), samples and controls were added in duplicate, then incubated for 90 min at room temperature. Plates were washed with 0.05% Tween 20 in phosphate buffered saline (PBS), anti-Human IgG-HRP conjugate was added and the plates were then incubated for 1 h. After the plates were washed with 0.05% Tween in PBS, SIGMA FAST OPD tablet (Sigma-Aldrich, P9187-50SET) diluted in purified water was added, and the OD was then measured at 450 nm. Samples from 2 year areas and 4 years areas were mixed and analysed together. Duplicate optical densities (ODs) of the ELISA results were averaged and plates were normalized using the positive control sample. The cut-off for seropositivity was defined as the mean OD plus three standard deviations obtained in twelve samples of negative controls sera from American malaria-naïve at the same concentration as the test samples.

#### Malaria parasitaemia

Thick blood smears were stained with 10% Giemsa for 15 min. Asexual parasite densities for *P. falciparum, Plasmodium malariae* and *Plasmodium ovale* were counted against 200 white blood cells (WBCs) and converted to parasites/μL assuming 8000 WBC/μL. A blood smear was considered to have negative results if no parasites were identified in 100 high-power fields. Slides were read by an experienced certified microscopist blinded to the treatment allocation. 10% of slides were re-read by a blinded expert reader for quality control.

### Statistical analysis

Data were entered and verified using DataFax. ELISA data were directly exported for analysis in StatView Version 5.0.1.0 (SAS Institute Inc) and Stata (version 14). Antibody seropositivity was considered the primary endpoint for this study, and antibody levels were treated as secondary endpoints. Proportions were compared using Chi square test. ELISA OD levels were compared between groups using Kruskal–Wallis test. Logistic regression was used to compare antibody seropositivity between groups of children who received SMC for 4 years and those who received it for 5 years, adjusting for age gender and malaria infection. p-values less than or equal to 0.05 were considered significant.

## Results

### Study population characteristics

A total of 461 children aged between 46–65 months were enrolled, 242 children in areas where SMC had been implemented for 4 years and 219 children in areas where SMC had been implemented for 2 years (Table 1). There were no differences in age or gender distribution between groups at the time that blood samples were collected. Prevalence of malaria infection was significantly higher in children who received SMC for 2 years compared to those who had received SMC for 4 years (33.1% versus 19.1%, p = 0.001).

**Table 1:** Baseline characteristics of the study children

	SMC 2 years (n = 242)	SMC 4 years (n = 219)	p
Age (months)			
Median (min–max)	54 (46–65)	54 (46–65)	0.84
Gender			
Male (%)	49.1	51.9	0.56
Parasite prevalence (%)	33.1	19.1	0.001

### Antibody prevalence

Prevalence of seropositivity to three malarial antigens in children who received SMC for 2 years or for 4 years are presented in Table 2. Anti-AMA1 seroprevalence was 88.8% in children who received SMC for 2 years and 95.3% in children who received SMC for 4 years, and this difference was significant (age, gender and parasitaemia adjusted odd ratio (aOR) = 3.16, 95% CI 1.44–6.95, p = 0.004). Anti-MSP-1_42_ seroprevalence was similar in children in the areas where SMC had been implemented for 2 years and in those resident in areas where SMC had been implemented for 4 years; (aOR = 1.06, 95% CI 0.62–1.80, p = 0.80). Anti-CSP seroprevalence was 81.9% in children who received SMC for 2 years and significantly higher (91.2%) in children exposed to SMC for 4 years (aOR = 3.14, 95% CI 1.70–5.76, p < 0.001).

**Table 2:** Prevalence of seropositivity to three malarial antigens in children who received seasonal malaria chemoprevention (SMC) for 2 years or for 4 years

	n	%	95% CI	Unadjusted			Adjusted^a^		
				OR	95% CI	p	OR	95% CI	p
AMA1									
SMC 2 years	215	88.8	84.5–92.8	Ref	–	–	Ref	–	–
SMC 4 years	205	95.3	92.5–98.2	2.57	1.22–5.45	0.014	3.16	1.44–6.95	0.004
MSP-1_42_									
SMC 2 years	208	86.0	81.5–90.4	Ref	–	–	Ref	–	–
SMC 4 years	183	85.1	80.3–90.0	0.93	0.55–1.58	0.8	1.06	0.62–1.80	0.8
CSP									
SMC 2 years	196	81.9	76.0–86.0	Ref	–	–	Ref	–	–
SMC 4 years	197	91.2	87.0–95.4	2.57	1.43–4.59	0.001	3.14	1.70–5.76	< 0.001

SMC 2 years = received SMC for 2 years; SMC 4 years = received SMC for 4 years

^a^adjusted for age, gender and malaria infection

### Antibodies levels

**Fig. 1:**
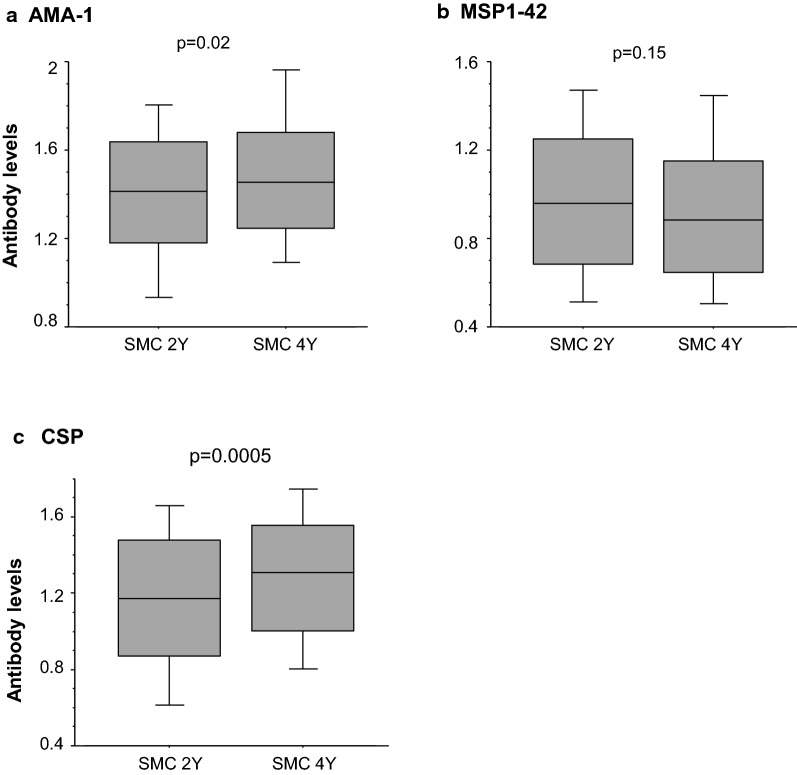
Antibody levels expressed as optic density in children who received SMC for 2 years and children and in those who received SMC for 4 years. The number of children in each group are as follow: SMC 2 years, n = 242 and SMC 4 years, n = 219

Antibody levels to AMA1, MSP-1_42_ and CSP in children who received SMC for 2 years and children who received SMC for 4 years are presented in Fig. 1. Median IgG levels to AMA1 and to CSP were significantly lower in children who received SMC for 2 years compared to those who received SMC for 4 years (1.41 (IQR: 1.17–1.64) and 1.45 (IQR: 1.24–1.68) p = 0.02 and 1.17 (IQR: 0.87–1.47) and 1.30 (IQR: 1.00–1.56) p = 0.0005, respectively). The median IgG levels to MSP-1_42_ were similar between the two groups (0.95 (IQR: 0.68–1.15) and 0.88 (IQR: 0.64–1.15), p = 0.15).

### Discussion

Whether the implementation of SMC over a long period of time impairs the acquisition of protective immune responses to malaria, thereby increasing the risk of disease in later years, has been a concern. In the previous study [10], the exposure to SMC reduced antibody levels to AMA1, MSP-1_42_ and CSP, but the duration of exposure to SMC from one to 3 years, had no effect on antibody levels to these antigens. The current study evaluated the effect of four consecutive years of SMC on acquisition of IgG antibodies to malarial antigens in children who had received SMC for 4 years compared to those who had received SMC for 2 years. It has been long known that naturally acquired immunity to malaria can clear parasites and prevent severe disease. However, the targets of protective immunity are unknown. In the current study, antibody levels to AMA-1, MSP-1 and CSP are used as markers of immune response to malaria exposure. The study showed that the prevalence and levels of IgG antibodies to the three proteins were not significantly lower in children who had received SMC for 4 years compared to those who had received SMC for 2 years. The seroprevalence was even significantly higher for AMA1 and CSP antibodies in the 4 years group, although the absolute differences were less than 10%. IgG levels to AMA1, MSP-1_42_ and CSP followed the same patterns. It is possible that the children in the group who received SMC for two year group had more exposure to malaria earlier in life before they received SMC and that this had in some way down-regulated the immune response. In a study in The Gambia, children who have had seasonal malaria chemoprophylaxis with pyrimethamine and dapsone for three years had higher cell-mediated immune responses to malaria antigens, than the control children who did not receive seasonal malaria chemoprophylaxis [16].

The high proportion of seropositivity to AMA1, MSP-1_42_ and CSP in this study suggests that malaria exposure remains substantial despite SMC. Consistent with results from previous surveys conducted in 2016 in the same area [10], about one out of five children carried malaria parasitaemia in the areas where SMC had been implemented for 4 years compared to about one third of those in the areas where SMC was implemented for 2 years. The lower prevalence of malaria in children in areas where SMC was implemented for 4 years may reflect a larger effect of a cumulative reduction in the transmission in these areas. In Senegal, SMC was associated with reduction in transmission as shown by a reduced incidence of confirmed malaria in children and adults above the age for SMC, who did not receive SMC in the areas where SMC was used compared to those in the area where it is not [17]. Malaria parasitaemia and past malaria morbidity were shown to be strongly associated with a higher specific IgG response in a study in Senegal [18]. Lower prevalence of MSP-1_19_ seropositivity was reported in children who received biannual azithromycin distribution compared to whose who received annual distribution in Niger [9]. In Ugandan children, sustained clinical protection and higher IL-2 and TNF-α production were reported in children with high adherence to monthly chemoprevention with dihydroartemisinin-piperaquine [8].

The finding in this study are consistent with previous report in the same areas [10] and in Senegal [7] suggesting that long-term malaria chemoprevention with SMC with SP + AQ has a limited impact on the development of antibody response against *P. falciparum* antigens. However, in all these studies including this one, differences in other factors such as malaria transmission including geographical-based heterogeneity, use of other preventive measures such as insecticide-treated nets and treatment-seeking behaviours between the study areas could not be excluded and constitute a limitation of these studies. In the current study, children who have never received SMC could not be found and used as control due to nation wide implementaton of SMC starting 2016. However, the consistency with the previous reports in studies with controls who had not received SMC, was reassuring. Strengths of this study include the fact that the samples were collected at the same time in children in both areas with limited age range of 46 to 65 months and analysed together.

## Conclusion

Four years of administration of anti-malarials through SMC was not associated with a reduction in IgG levels to AMA1, MSP-1_42_ and CSP compared to levels found in children who had received SMC for only 2 years suggesting that it is unlikely that these children will be at increased risk of malaria when they stop receiving SMC at the age of 5 years.

## Data Availability

The corresponding author had full access to all the data in the study and data are available at request to the corresponding author.

## Abbreviations

AMA-1: Apical membrane antigen 1
AQ: Amodiaquine
CSP: Circumsporozoite protein
ELISA: Enzyme-linked immunosorbent assay
GLURP: Glutamate-rich protein
CI: Confidence intervals
IgG: Immunoglobulin G
MRTC: Malaria research and training center
SMC: Seasonal malaria chemoprevention
MSP-1: Merozoite surface protein 1
OD: Optical density
OR: Odd ratio
SP: Sulfadoxine–pyrimethamine
